# Comprehensive Profiling of Hypoxia-Related miRNAs Identifies miR-23a-3p Overexpression as a Marker of Platinum Resistance and Poor Prognosis in High-Grade Serous Ovarian Cancer

**DOI:** 10.3390/cancers13133358

**Published:** 2021-07-04

**Authors:** Paola Todeschini, Elisa Salviato, Chiara Romani, Vittoria Raimondi, Francesco Ciccarese, Federico Ferrari, Germana Tognon, Sergio Marchini, Maurizio D’Incalci, Laura Zanotti, Antonella Ravaggi, Franco Odicino, Enrico Sartori, Donna M. D’Agostino, Michele Samaja, Chiara Romualdi, Eliana Bignotti

**Affiliations:** 1 Angelo Nocivelli’ Institute of Molecular Medicine, ASST Spedali Civili di Brescia, University of Brescia, 25121 Brescia, Italy; todeschini.paola@gmail.com (P.T.); chiara.romani@unibs.it (C.R.); laura.zanotti@unibs.it (L.Z.); antonella.ravaggi@unibs.it (A.R.); 2 Division of Obstetrics and Gynecology, ASST Spedali Civili di Brescia, 25123 Brescia, Italy; federicogferrari@gmail.com (F.F.); germanatognon@gmail.com (G.T.); franco.odicino@unibs.it (F.O.); enrico.sartori@unibs.it (E.S.); 3 Department of Biology, University of Padua, 35122 Padua, Italy; elisa.salviato.2@phd.unipd.it (E.S.); chiara.romualdi@unipd.it (C.R.); 4 Istituto Oncologico Veneto IOV—IRCCS, 35128 Padua, Italy; vittoria.raimondi@iov.veneto.it (V.R.); francesco.ciccarese@iov.veneto.it (F.C.); dm.dagostino@unipd.it (D.M.D.); 5 Department of Clinical and Experimental Sciences, Division of Obstetrics and Gynecology, University of Brescia, 35122 Brescia, Italy; 6 Department of Oncology, Istituto di Ricerche Farmacologiche Mario Negri, IRCCS, 20156 Milan, Italy; sergio.marchini@marionegri.it (S.M.); maurizio.dincalci@marionegri.it (M.D.); 7 Department of Biomedical Sciences, University of Padua, 35131 Padua, Italy; 8 Department of Health Science, University of Milan, 20122 Milan, Italy; michele.samaja@unimi.it

**Keywords:** miRNA, hypoxia, ovarian cancer, platinum response, prognostic marker, miR-23a-3p, APAF1

## Abstract

**Simple Summary:**

In the present paper, we identified miR-23a-3p, a hypoxia regulated-microRNA (miRNA), as a potential biomarker of chemoresistance and poor outcome in two independent cohorts of high-grade serous ovarian carcinoma (HGSOC) patients. Then, we predicted the involvement of miR-23a-3p in the platinum resistance pathway, together with its target APAF-1 gene, and validated their anticorrelation and association with platinum response in HGSOC patients and cell lines. We propose that the evaluation of miR-23a-3p expression may provide important clinical indications on patients not responding to platinum treatment and that the miR23a-3p/APAF1 axis could be considered a possible target for personalized medicine in HGSOC patients.

**Abstract:**

The onset of chemo-resistant recurrence represents the principal cause of high-grade serous ovarian carcinoma (HGSOC) death. HGSOC masses are characterized by a hypoxic microenvironment, which contributes to the development of this chemo-resistant phenotype. Hypoxia regulated-miRNAs (HRMs) represent a molecular response of cancer cells to hypoxia and are involved in tumor progression. We investigated the expression of HRMs using miRNA expression data from a total of 273 advanced-stage HGSOC samples. The miRNAs associated with chemoresistance and survival were validated by RT-qPCR and target prediction, and comparative pathway analysis was conducted for target gene identification. Analysis of miRNA expression profiles indicated miR-23a-3p and miR-181c-5p over-expression as associated with chemoresistance and poor PFS. RT-qPCR data confirmed upregulation of miR-23a-3p in tumors from chemoresistant HGSOC patients and its significant association with shorter PFS. In silico miR-23a-3p target prediction and comparative pathway analysis identified platinum drug resistance as the pathway with the highest number of miR-23a-3p target genes. Among them, APAF-1 emerged as the most promising, being downregulated in platinum-resistant patients and in HGSOC chemo-resistant cells. These results highlight miR-23a-3p as a potential biomarker for HGSOC platinum response and prognosis and the miR23a-3p/APAF1 axis as a possible target to overcome platinum-resistance.

## 1. Introduction

**Figure 1:**
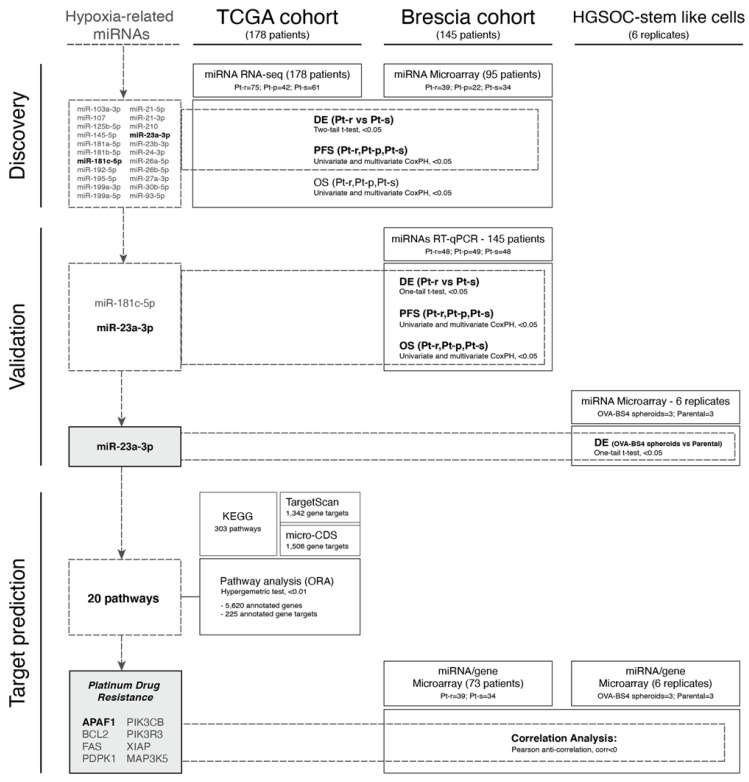
Workflow of the study.

Ovarian carcinoma (OC) represents the fifth leading cause of cancer death among women worldwide [1], with high-grade serous ovarian carcinoma (HGSOC) the most common and aggressive histological type. HGSOC develops rapidly and is frequently diagnosed at an advanced stage when multiple synchronous tumor lesions are localized to the ovary, as well as in other anatomical sites within the peritoneal cavity [2]. Resistance to chemotherapy is one of the major challenges in HGSOC clinical management since, after a positive initial response to primary treatment, most patients develop platinum-resistant recurrence, a lethal disease for which effective therapies do not exist [2]. The rapid expansion of HGSOC tumor masses requires the presence of a vascular network supplying oxygen and nutrients essential for their growth. However, when tumor cell proliferation exceeds angiogenesis, the highly abnormal microvasculature fails to cover the oxygen requirement, and tumor cells are exposed to an O2-deficient environment [3]. This persistent hypoxic condition induces changes in HGSOC gene expression that promote cancer progression, invasion as well as resistance to chemotherapy and the “angiogenic switch”, through transcriptional activation of the pro-angiogenic vascular endothelial growth factor (VEGF) gene [4,5]. Consequently, angiogenesis is considered an attractive target for ovarian cancer therapy with the anti-VEGF antibody Bevacizumab approved in the first-line setting, in addition to platinum and taxane combination, for patients with advanced-stage OC [6,7], as well as second-line treatment in combination with platinum and gemcitabine [8]. However, no molecular biomarkers predictive of response to Bevacizumab currently exist, and patients are still selected for the drug only on the basis of their clinical characteristics (including stage, debulking status and presence of ascites). Over the past few years, protein-coding hypoxia-regulated genes have been joined by specific miRNAs, thus adding a new paradigm of gene expression regulation to an already complex process and providing an additional link between tumor-specific stress conditions and gene expression control. The first evidence for a hypoxic cancer-related miRNA signature was described by Kulshreshtha et al., who reported a group of “hypoxia-regulated miRNAs” (HRMs), dysregulated in response to low oxygen tension in breast and colon cancer cell lines and involved in cancer cell survival in a stressful microenvironment [9]. In the present study (Figure 1), we investigated the role of HRMs in platinum response and prognosis in HGSOC patients by analyzing the expression of a panel of selected HRMs in a large collection of TCGA samples [10] and in samples from patients treated at ASST Spedali Civili di Brescia (Brescia, Italy). We identified and validated miR-23a-3p over-expression as associated with platinum resistance and worse prognosis. Pathway analyses, complemented by computationally predicted miRNA-gene interactions, identified a set of target genes involved in chemotherapy response and in particular APAF-1 gene, whose functional interactions with miR-23a-3p are likely to have an important role in HGSOC cell survival.

## 2. Materials and Methods

### 2.1. Selection of Candidate Hypoxia-Related miRNAs

Ovarian cancer-specific miRNAs related to hypoxia were selected from the literature following a Medline search using the MeSH terms ‘ovarian cancer’/‘ovarian carcinoma’ and ‘microRNA’/‘miRNA’ and ‘hypoxia’ [11,12,13,14,15,16,17,18] and from the study of Kulshreshtha et al. [9].

### 2.2. Patient Sample Cohorts

A total of 323 HGSOC tumor samples were gathered from two independent tumor tissue collections: 145 from the Brescia cohort and 178 catalogued in TCGA (Table 1 and Supplementary Methods, Section S1). Based on the time interval between the end of first-line chemotherapy and relapse (platinum-free interval, PFI), patients from the two cohorts were classified into three groups: (i) platinum-sensitive (Pt-s, PFI > 12 months), (ii) platinum-partially sensitive (Pt-ps, PFI within 6–12 months from the last round of chemotherapy), and (iii) platinum-resistant (Pt-r, PFI< 6 months) [19]. TCGA patients for whom PFI information was not available (due to incomplete or missing annotations) were excluded from the analysis. The study on the Brescia cohort was performed following the Declaration of Helsinki set of principles and approved by the Research Review Board—the Ethics Committee—of the ASST-Spedali Civili, Brescia, Italy (study reference number: NP1676). Written informed consent was obtained from all patients enrolled.

### 2.3. Tissue Sample Collection and RNA Extraction

Tissue sample collection and RNA extraction were performed as previously described [20] and detailed in Supplementary Methods, Section S2.

### 2.4. miRNA and Gene Expression Profiles

Gene and miRNA microarray experiments were carried out on 95 HGSOC tissue samples from the Brescia cohort, as previously described [20,21] and detailed in Supplementary Methods, Section S3.

### 2.5. Validation by RT-qPCR

The miRNA validation using RT-qPCR was performed on 145 HGSOC tissue samples from the Brescia cohort, as previously described [22] and detailed in Supplementary Methods, Section S4.

### 2.6. miRNA Normalization Strategy

To accurately quantify miRNA levels, a reliable normalization relative to an endogenous miRNA is mandatory [23]. To identify stable miRNAs acting as normalizers for RT-qPCR expression data, we combined data from the literature with those resulting as invariant in our cohort of HGSOC microarray data, as detailed in Supplementary Methods, Section S5.

### 2.7. Cell Line Transfection and Apoptotic Cell Death Detection

All the materials and methods regarding silencing and overexpressing miR-23a-3p in HGSOC cell line models are reported in Supplementary Methods, Section S8–S13.

### 2.8. Statistical Analysis

All statistical analyses were performed using the R software (R Foundation for Statistical Computing, Vienna, Austria; version 3.4.0).

#### 2.8.1. Pre-Processing and Differential Expression Analysis

The miRNA counts (TCGA, see Supplementary Methods, Section S1) were used to test for differential expression in RNA-seq experiments by use of the negative binomial distribution (DEseq2 package, version 1.18.1) [24]. Quantile normalization (limma package, version 3.34.9) [25], after log-transformation (base 2) of pseudo-counts, was used for survival analysis. Log-transformed (base 2) microarray data (Brescia cohort, see Supplementary Methods, Section S1)—for both miRNA and mRNA expression profiles—were normalized using the loess method, and the empirical Bayes test (limma package, version 3.34.9) [25] was performed for the differential expression analysis.

The miRNA expression profiles of cells with stemlike characteristics (OVA-BS4 spheroids), derived from a primary human HGSOC cell line as previously described [26] and detailed in Supplementary Methods Sections S6 and S7, were normalized with quantile normalization, after log-transformation of probes intensities. Normalized matched mRNA profiles were downloaded from ArrayExpress (E-MTAB-4799) [26].

#### 2.8.2. RT-qPCR Data and Equivalence Analysis

RT-qPCR data from different groups were compared using a *t*-test for raw Cq and delta-normalized values (two- and one-tail, respectively). Equivalence of reference candidates between Pt-s and Pt-r patients was assessed using the two one-sided test (TOST) approach (equivalence package, version 0.7.2) [27], with the adoption of the default confidence level (alpha = 0.05). To ensure adequate power of the test, the equivalence range (±ε) was chosen depending on data variability following Wellek criteria [28].

#### 2.8.3. Survival Analysis

The associations between miRNA profiles and survival outcomes were assessed with the Cox proportional hazard (CoxPH) model (survival R package, version 2.43–3), both in univariate and multivariate analysis. Patients were stratified into groups based on quartiles of the normalized (−∆Cq) expression level distribution. Overall Survival (OS) was defined as the time from the date of diagnosis to the day of death or last follow-up. Progression Free Survival (PFS) was defined as the time from the date of diagnosis to the date of the first recurrence/progression or last follow-up.

#### 2.8.4. Over-Representation Analysis

The microT-CDS (version 5.0) [29] and TargetScan (release 7.2) [30] web servers were used to identify predicted interactions between miRNA-23a-3p and target genes. Only miRNA-mRNA interactions with miTG scores greater than 0.7 and context++ scores less than 0 (default parameters) were selected (1506 and 1342, respectively). In order to assess whether a certain biological pathway was significantly enriched for a certain miRNA, we performed an over-representation analysis (ORA) based on the hypergeometric test, as proposed by Backes et al. [31]. KEGG pathway annotations were retrieved using graphite R package (version 1.28.2) [32]. The *p*-values were computed using as background the number of validated targets (225) over the total number of annotated genes (5620) for the entire collection of considered pathways (303). Bonferroni corrections were applied to account for multiple testing. Information on experimentally supported miRNA targets was retrieved from DIANA-TarBase v8 [33].

## 3. Results

### 3.1. Patient Cohort Description

The clinicopathologic characteristics of 145 HGSOC patients belonging to the Brescia cohort and 178 patients from TCGA cohort are summarized in Table 1. All of the patients in the Brescia cohort were diagnosed with high-grade serous histological type and staged according to FIGO guidelines as stage III (77%) or IV (23%). Patients were followed from the date of surgery until death or the latest record retrieved, August 2018 (median follow-up, 3.6 years; range, 0–15 years). The median age at diagnosis was 62, and the median overall survival (OS) time was 43.7 months (range 1.2–177.3 months). Similarly, all 178 TCGA patients were diagnosed with high-grade serous histological type and staged according to FIGO guidelines as stage III (89%) or IV (11%). Patients were followed from the date of surgery until death or the latest record retrieved (median follow-up, 2.62 years; range, 0–12.7 years). The median age at diagnosis was 60, and the median overall survival (OS) time was 33.3 months (range 0.8–152.9 months).

### 3.2. Selection of Hypoxia-Related miRNAs

A Medline search was performed to identify HRMs with a potential role in HGSOC, and eight articles were selected [11,12,13,14,15,16,17,18]. A total of 8 ovarian cancer-specific miRNAs related to hypoxia and selected from the abovementioned Medline search [11,12,13,14,15,16,17,18], together with 20 HRMs reported by Kulshreshtha et al. [9], were included in the study (see Supplementary Results, Table S1). Three miRNAs were in common between the 2 groups; hence, a total of 25 miRNAs were selected for further analysis.

### 3.3. Evaluation of HRMs in HGSOC Datasets

Expression levels of the 25 selected miRNAs were derived from microarray data of 95 HGSOC patients of the Brescia cohort [20] and from published RNA Seq data of 178 TCGA patients (TCGA/OV project, see Supplementary Methods), and correlated with response to treatment and prognosis. Three miRNAs (miR-106a-3p, miR-138 and miR-630) were discarded because they were undetected in RNA-seq data, thus yielding 22 miRNAs for further analysis. HGSOC patients were classified as ‘Pt-r’ or ‘Pt-s’ based on their response to carboplatin treatment in the Brescia and TCGA cohorts (73 and 136 samples, respectively; see Materials and Methods), and the expression of the 22 miRNAs was evaluated by comparing the 2 groups. Results showed that miR-181c-5p and miR-23a-3p were significantly over-expressed in platinum-resistant patients in both cohorts (adjusted *p*-value ≤ 0.05, Table 2). The prognostic significance of the two miRNAs was assessed by univariate and multivariate models, accounting for age and residual tumor. As shown in Table 3, both miRNAs were found to be associated with PFS in the Brescia cohort, by both univariate and multivariate analysis (*p*-value ≤ 0.05), while neither miRNA was significantly associated with OS (Supplementary Results, Table S2).

### 3.4. Validation of miR-23a-3p and miR-181c-5p Expression by RT-qPCR

The miR-23a-3p and miR-181c-5p expression was assessed by RT-qPCR on 145 HGSOC tissues from the Brescia cohort (including the 95 samples already profiled for miRNA expression) and associated with platinum response and survival.

**Figure 2:**
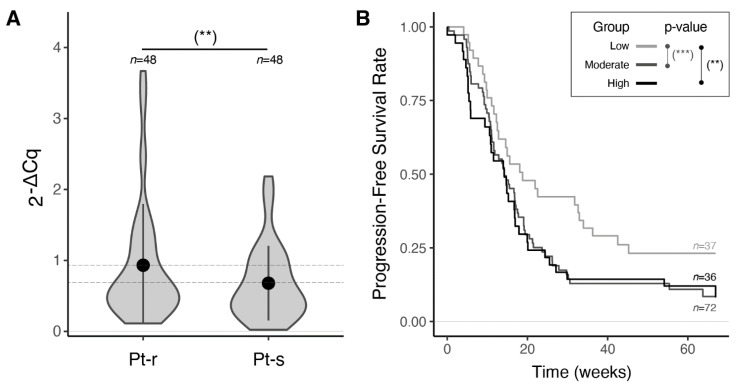
Prognostic performance of miR-23a-3p in the Brescia cohort. (**A**) Violin plots and error bars (mean +/− standard deviation) showing normalized miR-23a-3p expression levels measured by RT-qPCR in HGSOC samples from platinum-resistant patients (Pt-r) compared to platinum-sensitive patients (Pt-s) (*p*-value = 0.03, one-sided *t*-test). Complete results in Table 4. (**B**) Progression-free survival curves (Coxph model adjusted by residual tumor, age and Bevacizumab), stratified by quartiles of normalized miR-23a-3p expression levels (Q_1_: low; Q_2_–Q_3_: moderate; Q_4_: high). Complete results in Supplementary Results Table S4. (**) *p* ≤ 0.05, (***) *p* ≤ 0.01.

The miR-23a-3p was confirmed to be significantly up-regulated in Pt-r compared to Pt-s patients (*p*-value = 0.03, Table 4 and Figure 2A). Multivariate survival analysis, accounting for residual tumor age and Bevacizumab treatment response on the entire Brescia cohort, revealed miR-23a-3p over-expression as an independent predictor of worse OS and PFS (Table 5 and Supplementary Results, Table S3). As expected, a strong association of known prognostic factors, such as age and residual tumor, with both OS and PFS (*p*-value ≤ 0.01) was found (Supplementary Results, Table S3). Moreover, as reported in the literature [34,35], treatment with Bevacizumab was confirmed to have a beneficial effect only on PFS. Stratification of the patients based on miR-23a-3p expression yielded a clear separation of the PFS curves, with patients in the lowest quartile of expression showing significantly longer PFS than patients in the middle or highest expression quartiles (HR = 1.92, Supplementary Results, Table S4 and Figure 2B).

Notably, miR-23a-3p was prognostic for PFS even in the subgroup of 22 patients treated with Bevacizumab, when accounting for residual tumor and age (HR = 1.8, Supplementary Results, Table S5).

Due to its significant association with response to platinum and prognosis, we selected miR-23a-3p for further investigations.

### 3.5. miR-23a-3p Expression in Ovarian Carcinoma Stem-Like Cells

**Figure 3:**
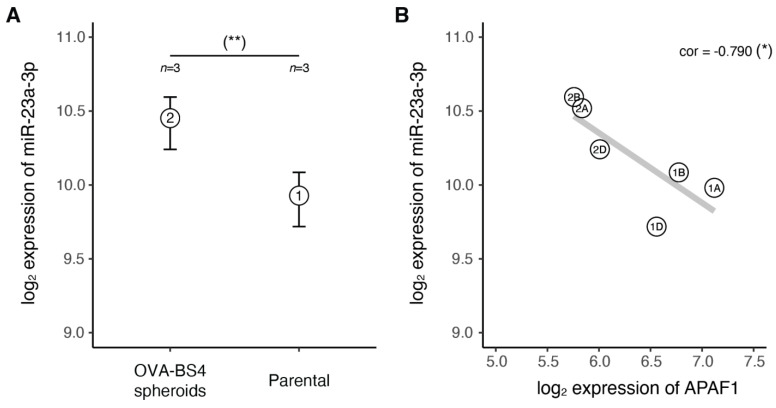
The miR-23a-3p in HGSOC-Stemlike cells. (**A**) Range (bars) and mean (points) for miR-23a-3p expression levels measured by microarray in stemlike cell replicates from OVA-BS4 spheroids (1) compared to the parental cell line replicates (2) (*p*-value = 0.013, one-tail *t*-test). (**B**) Anti-correlation between miR-23a-3p and APAF1 expression levels measured by matched miRNA/gene microarray (*p*-value = 0.061, Pearson’s product-moment). (*) *p* ≤ 0.1, (**) *p* ≤ 0.05.

As cancer stem cells (CSCs) have the fundamental property of being resistant to both chemotherapy and radiation, we tested whether miR-23a-3p might be deregulated in these cells. Our group recently isolated a population of cells with stemlike characteristics (OVA-BS4 spheroids) from a primary HGSOC cell line and characterized their miRNA and mRNA expression profiles by microarrays ([26] and Supplementary Methods, Section S7). Using these datasets, we evaluated the expression of miR-23a-3p in CSC cells compared to their parental lines (Figure 3A). In agreement with our hypothesis, we found miR-23a-3p resulted significantly up-regulated in the OVA-BS4 spheroid cell line compared to parental cells (*p*-value = 0.013, one-tail *t*-test).

### 3.6. In Silico miR-23a-3p Target Prediction and Comparative Pathway Analysis

We performed an in silico target analysis using two different algorithms (TargetScan and microT-CDS), resulting in a set of 753 common predicted targets (Jaccard index: 33.4%). We then performed ORA analysis comparing—for each pathway—the observed with the expected number of targets. Twenty out of 303 analyzed pathways (6.6%) were identified as significantly enriched (adjusted *p*-value ≤ 0.001). Interestingly, the ‘platinum drug resistance’ pathway showed the second most prominent enrichment (Table 6), suggesting the involvement of miR-23a-3p in this process.

To further investigate the link between miR-23a-3p and platinum drug resistance, we evaluated the expression correlation of miR-23a-3p with its eight predicted gene targets identified in the ‘platinum drug resistance’ pathway (Table 7). We explored miRNA-target correlation in two different datasets: (i) the matched HGSOC miRNA and gene expression profiles of the Brescia cohort [20,21], and (ii) the matched miRNA–mRNA dataset of microarray profiles of HGSOC-stemlike cells (OVA-BS4 spheroids) isolated from a primary HGSOC cell line [26] (Figure 3B). Assuming that increased miRNA expression induces target mRNA degradation and/or translational repression [36], we detected a consistent anticorrelation pattern (Pearson correlation) in both datasets only for the APAF1 gene (ρ = −0.227 and −0.79).

### 3.7. The miR-23a-3p/APAF1 Axis and Carboplatin Sensitivity

To functionally assess the miR-23a-3p-APAF1 interaction, we conducted a pilot in vitro study on an HGSOC primary cell line (OSPC2) and on the widely studied OC cell line OVCAR3, which express high and low miR-23a-3p levels, respectively (Figure S1). As described in the Supplementary Results, transfection of OSPC2 with a miR-23a-3p inhibitor resulted in an increase in APAF1 protein levels, while transfection of OVCAR3 cells with a miR-23a-3p mimic produced a reduction in APAF1 (Figures S2A,B and S3A,B). Further experiments provided evidence that miR-23a-3p suppresses apoptosis of tumor cells and confers platinum chemoresistance by regulating the expression of its direct target APAF1 (Figure S3C,D and Table S6). 

## 4. Discussion

Resistance to standard platinum-based chemotherapy is the principal cause of poor outcome in HGSOC patients [2]. Thus, the discovery of reliable biomarkers predictive of response is crucial to inform patient selection for effective and safe treatment strategies. In this study, starting from the hypothesis of the involvement of hypoxic miRNAs in a more aggressive HGSOC phenotype, we demonstrated that over-expression of miR-23a-3p correlates with chemo-resistant disease and is an independent biomarker of poor prognosis in HGSOC patients. Importantly, the role of this miRNA as a prognostic biomarker is independent from other well-recognized clinical characteristics, such as age and residual tumor. The association of miR-23a-3p expression with a more aggressive tumor phenotype has been previously reported in OC, both in tissues and in cell lines [37,38,39,40,41,42,43]. However, to our knowledge, this is the first study assessing miR-23a-3p expression by three different technologies in two independent cohorts of OC patients, clinically homogeneous regarding histotype (serous), tumor grade (high), stage (advanced) and treatment (first-line platinum-based chemotherapy). This result provides novel indications for the management of newly diagnosed HGSOC patients, by identifying women who may not benefit from platinum therapy and may be directed to alternative treatments. In addition, the evaluation of miR-23a-3p expression at relapse could help in identifying patients unlikely to respond to platinum who can be more efficiently treated with other regimens, avoiding the toxicity of unnecessary therapies. It is worth noting that miR-23a-3p over-expression retained its role as a marker of poor prognosis in the subgroup of patients treated with Bevacizumab, an anti-VEGF antibody that has been added to the OC standard of care first and second-line therapy regimens. The identification of biomarkers able to guide Bevacizumab treatment is still a pending issue, and our preliminary results indicate that miR-23a-3p might help to select OC patients who will benefit from antiangiogenic therapy. Consistent with our hypothesis of a role for miR-23a-3p in chemoresistance, we found that ‘platinum drug resistance pathway’ was one of the most enriched KEGG pathways for its mRNA targets. Evaluating the relationship of miR-23a-3p with the target mRNAs annotated in this pathway and employing an internal collection of gene expression profiles derived from HGSOC samples and cell lines [20,21], we found APAF1 as the experimentally validated and more anticorrelated miR-23a-3p target. Of note, miR-23a-3p over-expression together with APAF-1 downregulation characterized both platinum-resistant tumors and the ovarian carcinoma-stem-like cells (OVA-BS4 spheroids), previously reported by our group as highly resistant to platinum treatment. In addition, through a pilot in vitro study, we observed that by inhibiting miR-23a-3p expression, OSPC2 cells increase APAF1 levels, becoming more sensitive to carboplatin treatment, through an increase in apoptosis. On the contrary, enhanced miR-23a-3p levels caused a reduction in APAF1 expression and decreased cell death in the OVCAR3 cells, thus inducing a platinum-resistant phenotype.

APAF1, the apoptotic protease-activating factor 1, is a key component of the intrinsic apoptotic pathway. Indeed, carboplatin causes DNA damage that induces apoptosis, and one of the potential mechanisms implicated in platinum resistance is the inhibition of apoptosis [44]. Previously reported evidence for the role of miRNAs in regulating APAF1 expression in the context of ovarian cancer exists. In particular, Yeung et al. showed that exosomal transfer of stroma-derived miR-21 to OC cells confers chemoresistance and an aggressive phenotype through binding to the APAF1 mRNA [44]. Additionally, Eoh et al. reported that an miR-630 inhibitor attenuated chemo-resistant OC proliferation and invasion, likely by targeting APAF1, re-sensitizing cells to chemotherapy [45]. The involvement of the miR-23a-3p/APAF1 axis has been reported in several solid cancers, including colorectal, pancreatic and laryngeal carcinomas and glioma [37,46]. However, to our knowledge, this is the first study demonstrating the implication of the miR-23a-3p/APAF1 axis in carboplatin resistance of HGSOC cells.

## 5. Conclusions

Our results indicate that miR-23a-3p could be considered a candidate biomarker to direct platinum therapy in HGSOC patients in the first line setting and in the subsequent lines of therapy. Additional studies in independent multicentric cohorts are needed to confirm the prognostic value of miR-23a-3p. Furthermore, strategies based on the upregulation of APAF1 might be explored as a novel therapeutic target and a tool to resensitize HGSOC cells to carboplatin treatment.

## Figures and Tables

**Table 1:** Clinicopathological characteristics of HGSOC patients in the Brescia and TCGA cohorts.

Clinical Annotations	Brescia Cohort	TCGA Cohort
	No. of Patients	
**Total No. of patients**	145	178
**Age**		
Median (range) years	62 (36–85)	60 (35–88)
**Histotype**		
Serous	145 (100%)	178 (100%)
**FIGO Classification**		
III	112 (77%)	159 (89%)
IV	33 (23%)	19 (11%)
**Residual Tumor (RT)**		
RT = 0	41 (28%)	44 (25%)
RT > 0	104 (72%)	134 (75%)
**Treatment**		
Carboplatin + Paclitaxel	123 (85%)	169 (95%)
Carboplatin + Paclitaxel + Bevacizumab	22 (15%)	9 (5%)
**Platinum Status**		
**Carboplatin + Paclitaxel**		
Sensitive	48 (33%)	60 (34%)
Partially Sensitive	23 (16%)	41 (23%)
Resistant	48 (33%)	68 (38%)
NA	4 (3%)	-
**Carboplatin + Paclitaxel + Bevacizumab**		
Sensitive	7 (5%)	1 (0.5%)
Partially Sensitive	6 (4%)	1 (0.5%)
Resistant	9 (6%)	7 (4%)
**Median follow-up, years (range)**	3.6 (0–15)	2.62 (0–12.7)
Median PFS, months (range)	23.7 (1.7–172.6)	15.3 (0.8–111.7)
Median OS, months (range)	43.7 (1.2–177.3)	33.3 (0.8–152.9)

**Table 2:** Differential expression analysis. Differential expression analysis for 22 miRNAs in platinum-resistant (Pt-r) compared to platinum-sensitive (Pt-s) patients.

miRNA Name	Brescia Cohort—Microarray 73 Samples (39 Pt-r, 34 Pt-s)			TCGA Cohort—RNA-seq 136 Samples (75 Pt-r, 61 Pt-s)		
	Log 2(FC)	AveExpr	Adj. *p*-Value	Log 2(FC)	Mean	Adj. *p*-Value
hsa-miR-103a-3p	0.125	9.956	0.358	0.174	44316	0.324
hsa-miR-107	0.058	9.317	0.682	0.131	152.4	0.323
hsa-miR-125b-5p	0.493	10.508	0.040 **	−0.195	10786.2	0.327
hsa-miR-145-5p	0.602	7.031	0.032 **	−0.169	3572.4	0.314
hsa-miR-181a-5p	0.403	7.724	0.034 **	−0.019	11434.2	0.915
hsa-miR-181b-5p	0.174	5.776	0.309	0.208	1374.8	0.228
**hsa-miR-181c-5p**	**0.547**	**4.896**	0.036 **	**0.650**	**223.2**	0.004 ***
hsa-miR-192-5p	−0.082	4.712	0.740	0.241	223.4	0.230
hsa-miR-195-5p	−0.173	7.756	0.475	0.187	6.7	0.351
hsa-miR-199a-3p	0.684	9.412	0.027 **	−0.064	618.8	0.740
hsa-miR-199a-5p	0.856	7.786	0.012 **	−0.308	770.8	0.161
hsa-miR-21-5p	0.065	13.243	0.759	0.221	11189.6	0.268
hsa-miR-21-3p	0.091	7.215	0.703	−0.182	2141.6	0.305
hsa-miR-210	0.023	8.001	0.910	−0.035	1574.9	0.866
**hsa-miR-23a-3p**	**0.363**	**10.310**	0.007 ***	**0.286**	**6830.0**	0.033 **
hsa-miR-23b-3p	−0.177	9.554	0.389	0.183	4070.9	0.266
hsa-miR-24-3p	−0.008	10.460	0.945	0.252	3371.5	0.058 *
hsa-miR-26a-5p	0.188	10.206	0.138	−0.081	1902.6	0.572
hsa-miR-26b-5p	0.106	9.162	0.480	−0.015	265.4	0.917
hsa-miR-27a-3p	0.440	10.151	0.002 ***	0.193	1397.1	0.208
hsa-miR-30b-5p	−0.313	9.202	0.089 *	0.140	186.4	0.429
hsa-miR-93-5p	−0.238	8.468	0.109	0.051	16511.6	0.750

Adjusted *p*-value (FDR). * <0.1 ** <0.05 *** <0.01. Highlighted in bold: significant miRNAs (*p* value ≤ 0.05) in both cohorts.

**Table 3:** Progression-free survival analysis. Univariate and multivariate Progression-free survival (PFS) analysis (Coxph) for miR-23a-3p and miR-181c-5p expression in both cohorts.

miRNA Name	Univariate			Multivariate ^(1)^		
	Hazard	SE	*p*-Value	Hazard	SE	*p*-Value
**Brescia Cohort—95 samples**						
**hsa-mir-23a-3p**	1.819	0.250	0.017 **	2.003	0.257	0.007 ***
**hsa-mir-181c-5p**	1.349	0.111	0.007 **	1.268	0.113	0.037 **
**TCGA Cohort—178 samples**						
hsa-mir-23a-3p	1.072	0.112	0.539	1.076	0.113	0.517
hsa-mir-181c-5p	1.026	0.070	0.715	1.036	0.072	0.627

^(1)^ Multivariate model accounted for age and residual tumor (2 classes: RT = 0 and RT > 0). ** <0.05 *** <0.01. Highlighted in bold: significant miRNAs (*p* value ≤ 0.05).

**Table 4:** Differential expression analysis. The miR-23a-3p and miR-181c-5p expression in Pt-r (*n* = 48) and Pt-s (*n* = 48) patients evaluated by RT-qPCR (Brescia cohort).

miRNA Name	Mean (sd) Cq		Mean (sd) −ΔCq ^(1)^		Cq		−ΔCq ^(1)^	
	Pt-r	Pt-s	Pt-r	Pt-s	Stat (95%CI)	*p*-Value	Stat (95%CI)	*p*-Value
**hsa-miR-23a-3p**	24.82 (1.3)	25.39 (1.3)	−0.56 (1.0)	−1.04 (1.1)	−2.140 (−Inf, −0.128)	0.017 **	1.910 (0.063, Inf)	0.030 **
hsa-miR-181c-5p	30.61 (2.3)	31.09 (2.5)	−6.35 (2.1)	−6.73 (2.6)	−0.965 (−Inf, 0.345)	0.169	0.812 (0.404, Inf)	0.209

^(1)^ reference miR-16-5p. ** <0.05. One-side, T-test analysis. Highlighted in bold: significant.

**Table 5:** Overall and progression-free survival analysis. OS and PFS analysis (Coxph) for miR-23a-3p and miR-181c-5p expression evaluated by RT-qPCR in Brescia cohort samples (*n* = 145). Complete results for the multivariate analysis in Supplementary Results, Table S3.

miRNA Name	Univariate-ΔCq ^(1)^			Multivariate ^(2)^-ΔCq ^(1)^		
	Hazard (95% CI)	SE	*p*-Value	Hazard (95% CI)	SE	*p*-Value
**Overall Survival (OS)**						
**hsa-miR-23a-3p**	1.149 (0.977–1.352)	0.083	0.092 *	1.195 (1.027,1.390)	0.077	0.021 **
hsa-miR-181c-5p	1.026 (0.940–1.121)	0.045	0.561	1.025 (0.938,1.119)	0.045	0.587
**Progression-Free Survival (PFS)**						
**hsa-miR-23a-3p**	1.178 (1.016–1.366)	0.075	0.030 **	1.244 (1.071,1.446)	0.077	0.004 ***
hsa-miR-181c-5p	1.034 (0.957–1.118)	0.040	0.399	1.040 (0.960,1.127)	0.041	0.346

^(1)^ reference miR-16-5p; ^(2)^ Multivariate model accounted for residual tumor (2 classes: RT = 0 and RT > 0), age and Bevacizumab. * <0.10 ** <0.05 *** <0.01. Highlighted in bold: significant.

**Table 6:** Over-representation analysis of KEGG pathways for miR-23a-3p target genes. Differentially expressed pathways (Adjusted *p*-value ≤ 0.01).

Pathway		No. Genes	No. Targets	−log10 (adj. *p*-Value) ^(1)^	Target
**1**	Renal cell carcinoma	56	13	15.32	ARNT; ARNT2; CREBBP; CRK; EGLN2; GAB1; MET; PIK3CB; PIK3R3; PAK3; PAK6; TGFA; RAP1A
**2**	Platinum drug resistance	39	8	10	APAF1; BCL2; FAS; PDPK1; PIK3CB; PIK3R3; XIAP; MAP3K5
3	Hedgehog signalling pathway	47	8	8.21	CSNK1G1; CSNK1G3; CUL3; HHIP; SMURF2; SPOPL; BCL2; GSK3B
4	EGFR tyrosine kinase inhibitor resistance	79	11	7.41	BCL2; FGF2; GAB1; IL6R; JAK1; MET; PIK3CB; PIK3R3; PTEN; TGFA; GSK3B
5	ErbB signalling pathway	85	11	6.59	CRK; ERBB4; GAB1; PAK3; PAK6; PIK3CB; PIK3R3; TGFA; GSK3B; CBLB; STAT5B
6	Bacterial invasion of epithelial cells	53	7	5.57	CRK; GAB1; MET; PIK3CB; PIK3R3; WASL; DNM3
7	Non-small cell lung cancer	65	8	5.34	EML4; PDPK1; PIK3CB; PIK3R3; RXRG; TGFA; STAT5B; STK4
8	Glycosphingolipid biosynthesis—lacto and neolacto series	27	4	4.72	FUT4; FUT9; GCNT2; ST8SIA1
9	p53 signalling pathway	71	8	4.61	APAF1; BCL2; CCNG1; FAS; PTEN; RCHY1; SESN2; SESN3
10	mTOR signalling pathway	142	15	4.51	ATP6V1B2; ATP6V1C1; ATP6V1E1; FNIP2; FZD4; FZD5; GSK3B; LRP5; PDPK1; PIK3CB; PIK3R3; PTEN; SEH1L; SESN2; CHUK
11	Prostate cancer	85	9	4.25	CHUK; CREBBP; GSK3B; PDPK1; PIK3CB; PIK3R3; PTEN; TGFA; BCL2
12	Aldosterone-regulated sodium reabsorption	30	4	4.2	NEDD4L; PDPK1; PIK3CB; PIK3R3
13	Measles	107	11	4.14	CBLB; CHUK; JAK1; PIK3CB; PIK3R3; RCHY1; TNFAIP3; GSK3B; FAS; IL12B; STAT5B
14	Fc gamma R-mediated phagocytosis	91	9	3.66	ASAP1; CRK; PIK3CB; PIK3R3; PRKCE; WASL; CFL2; MARCKS; MARCKSL1
15	Adherens junction	69	7	3.62	CSNK2A2; MET; TGFBR2; WASL; YES1; TJP1; NLK
16	Small cell lung cancer	92	9	3.57	APAF1; BCL2; CHUK; COL4A4; PIK3CB; PIK3R3; PTEN; RXRG; XIAP
17	Mannose type O-glycan biosynthesis	23	3	3.49	CHST10; FUT4; FUT9
18	Non-alcoholic fatty liver disease (NAFLD)	71	7	3.42	FAS; IL6R; MAP3K5; PIK3CB; PIK3R3; GSK3B; CASP7
19	Phosphatidylinositol signalling system	86	8	3.1	DGKE; INPP5A; IPMK; PIK3C2A; PIK3CB; PIK3R3; PIP4K2B; PTEN
20	IL-17 signalling pathway	14	2	3.08	FOSB; TAB3

^(1)^ Bonferroni correction.

**Table 7:** Predicted miR-23a-3p targets involved in platinum drug resistance pathway. Reported are TargetScan, microT-CDS scores and Pearson correlations between matched mRNAs and miRNA microarray expression profiles in the Brescia cohort (73 samples: 39 Pt-r, 34 Pt-s) and OVA-BS4 cell line (6 biological replicates: 3 spheroids vs. 3 parental).

Symbol	Target Scan	microT−CDS	Tarbase	Pearson Correlation	
	Context Score	miTGscore	Experimentally Validated	Brescia Cohort Microarray	OVA−BS4 Cell Line Microarray
**APAF1**	**−0.54**	**0.96**	**Yes**	**−0.227**	**−0.790**
BCL2	−0.36	0.73	No	0.250	0.893
FAS	−0.51	0.84	No	0.124	−0.783
PDPK1	−0.15	0.92	Yes	−0.159	−0.002
PIK3CB	−0.17	0.91	Yes	0.040	−0.201
PIK3R3	−0.17	0.95	Yes	−0.126	0.927
XIAP	−0.28	0.95	No	0.052	−0.907
MAP3K5	−0.08	0.81	No	−0.038	0.816

Highlighted in bold: anti-correlated genes (<−0.1) in both considered datasets.

## Data Availability

The data presented in this study are openly available in EMBL-EBI ArrayExpress repository, reference number E-MTAB-7084 (miRNA microarray, Brescia cohort), E-MTAB-7083 (mRNA microarray, Brescia cohort), ‘pending number’ (miRNA microarray, HGSOC-stem like cells) and E-MTAB-4799 (mRNA microarray, HGSOC-stem like cells). Publicly available datasets were analyzed in this study. These data can be found in NIH GDC Data Portal, reference project TCGA-OV (miRNA RNA-seq, TCGA cohort).

## Supplementary Materials

The following are available online at https://www.mdpi.com/article/10.3390/cancers13133358/s1, Methods S1: Brescia and TCGA cohorts, Methods S2: Tissue collection and RNA extraction, Methods S3: miRNA and gene expression profiles, Methods S4: miRNA validation by reverse transcription-quantitative PCR (RT-qPCR), Methods S5: Selection of optimal reference miRNA for RT-qPCR data normalization, Methods S6: Characteristics of OVA-BS4 parental and OVA-BS4 spheroid cell lines, Methods S7: miRNA and gene expression profiles of OVA-BS4 parental and OVA-BS4 spheroid cell lines, Table S1: Characteristics of candidate miRNAs selected for RT-qPCR validation, Table S2: Brescia cohort—microarray Equivalence Test, Table S3: Brescia cohort-RT-qPCR. Equivalence Test, Methods S8: Cell lines and culture conditions, Methods S9: Cell transfection, Methods S10: Drug treatment, Methods S11: Apoptotic cell death detection, Methods S12: Apoptosis Analysis, Methods S13: RNA isolation from cell lines and RTqPCR, Methods S14: Western Blot, Results Table S1: Hypoxia-related miRNAs selected for expression analysis, Table S2: Overall survival analysis, Table S3: Complete results of Multivariate Survival Analysis, Table S4: Multivariate Progression-Free Survival Analysis (Coxph), Table S5: Multivariate Survival Analysis (Coxph) of the subgroup of 22 patients treated with bevacizumab, Table S6: Apoptosis cell death after carboplatin treatment in OSPC2 and OVCAR3 cell lines, Figure S1: miR-23a-3p expression in HGSOC cell lines by RTqPCR, Figure S2: miR-23a-3p expression modulation after transfection with inhibitor or mimic in HGSOC cell lines, Figure S3 upper panel. Immunoblots showing effect of miR-23a-3p modulation on APAF-1 expression before and after carboplatin treatment in HGSOC cell lines, Figure S3 bottom panel. Barplots representing apoptosis in HGSOC cell lines after transfection with miR-23a-3p inhibitor or mimic, followed by carboplatin treatment.
