# Supplementary material for: Comprehensive Profiling of Hypoxia-Related miRNAs Identifies miR-23a-3p Overexpression as a Marker of Platinum Resistance and Poor Prognosis in High-Grade Serous Ovarian Cancer
Source: Cancers (Basel). 2021 Jul 4;13(13):3358. doi: 10.3390/cancers13133358 (PMC8268862; doi:10.3390/cancers13133358)
Supplement: Supplementary file 1 [file cancers-13-03358-s001.zip › Todeschini_etal_SupplMethods_Cancers.pdf]

Supplementary Methods

1. Brescia and TCGA cohorts

We retrospectively collected a cohort of 145 cases of HGSOC diagnosed and treated at the Division of Obstetrics and Gynecology, ASST Spedali Civili di Brescia, University of Brescia, Italy, between November 2001 and August 2018. All patients were staged according to the International Federation of Gynaecologic and Obstetrics (FIGO) criteria and were classified as stage III/IV. Information regarding age, stage, macroscopic residual tumor after cytoreductive surgery (RT), response to chemotherapy and prognosis was recorded for all patients. From TCGA database (May 9, 2017) we downloaded miRNA Isoforms profiles (RNA-Seq) using GDC queries (TCGAbiolinks R library, version 2.6.12) for Ovarian Serous Cystadenocarcinomas (TCGA-OV project). Only those patients who received first-line carboplatin therapy were retained. Additional filter criteria were applied to race (white), stage (III and IV), grade (G3), and surgery procedure to ensure the comparability of the two collected cohorts. Table 1 summarizes the main clinico-pathological characteristics of the final cohort of 178 patients.

2. Tissue collection and RNA extraction

HGSOC tissues were obtained from chemotherapy-naïve patients at the time of primary surgery, snap-frozen in liquid nitrogen within 30 minutes and stored at -80°C until processing. Only samples containing at least 70% of tumor epithelial cells, as assessed by a staff pathologist, were used for further analysis. Total RNA was extracted from tissue samples using TRIzol® Reagent (Ambion), purified with RNeasy MinElute Cleanup® kit (Qiagen), with a modified protocol for co-purification of small RNAs according to the manufacturer's instructions. In detail, the starting sample volume was adjusted to 50 µl with RNase-free water, instead of 100 µl. Following the sequential addition of 350 µl of RLT buffer, 600 µl of 96% ethanol and 600 µl of 100% ethanol, the samples were processed as described in the RNeasy MinElute Cleanup Handbook (Qiagen). The RNA concentration and 260/280 absorbance ratio (A260/280) were measured with an Infinite M200 spectrophotometer (TECAN). RNA integrity was assessed with an RNA 6000 Nano LabChip kit and an Agilent 2100 Bioanalyzer (Agilent Technologies). RNA integrity number (RIN), generated with Agilent 2100 Expert software, was greater than 8 for all RNA samples.

3. miRNA and gene expression profiles

Microarray mRNA and miRNA expression experiments were performed on 95 HGSOC specimens using commercially available kits (G4851B Human Whole GE kit and G4470B Human miRNA kit, Agilent Technologies). According to manufacturer's instructions, RNA was labelled and hybridized as previously published (1) and the fluorescence intensity generated was measured with Agilent Feature Extraction software, version 11 (Agilent Technologies). In accordance with the MIAME guidelines, the array data files have been uploaded to the Array Express database (IDs EMTAB-7083 and E-MTAB-7084).

4. miRNA validation by reverse transcription-quantitative PCR (RT-qPCR)

Ten ng of purified RNA was reverse transcribed into cDNA following the manufacturer's instructions (miRCURY LNA Universal RT microRNA PCR system, Qiagen). The cDNA was diluted 1:50 and 4 µl were PCR-amplified using a CFX96 Touch™ Real-Time PCR Detection System (Bio-Rad), using iTaq Universal SYBR Green Supermix (Bio-Rad), and individual miRNA-specific PCR primer sets (miRCURY LNA miRNA PCR Assays, Qiagen) (Table S1).

An annealing temperature of 60° C and a total of 40 cycles were used for all primer pairs, and all reactions were run in triplicate. A melting curve was constructed for each primer pair to confirm amplification product specificity. Three inter-run calibration samples were used included on all plates to correct for the technical variance between the different runs and to compare results from different plates.

Table S1. Characteristics of candidate miRNAs selected for RT-qPCR validation.

miRNA	HGNC symbol	Seq. accession number	Target sequence length (nt)
miR-23a-3p	MIR23A	NR_029495	21
miR-181c-5p	MIR181C	NR_029613	22
miR-191-5p	MIR191	NR_029690	23
miR-26a-5p	MIR26A1	NR_029499	22
miR-16-5p	MIR16-1	NR_029486	22
miR-16-2-3p	MIR16-2	NR_029525	22
U6	RNU6-1	NR_004394	106

5. Selection of optimal reference miRNA for RT-qPCR data normalization

An extensive evaluation of published articles, focusing on the analysis of miRNA expression in HGSOC tissue samples, revealed miR-191-5p as a stably expressed miRNA (2). In addition, miR-26a-5p and miR-16-5p emerged as invariant in the Brescia cohort microarray data (Table S2). The expression levels of these three putative reference miRNAs were evaluated using RT-qPCR and tested for stability. The equivalence analysis (Table S3), preliminarily performed on a subset of 56 HGSOC samples, revealed miR-16-5p as the most uniformly expressed reference miRNA in the comparison between resistant and sensitive patients (p-value = 0.043) and in the entire dataset of 96 samples (p-value = 0.016). miR-16-5p was thus selected as the normalizer for relative quantification in our study.

6. Characteristics of OVA-BS4 parental and OVA-BS4 spheroid cell lines

The primary cell line OVA-BS4 was established after sterile processing of a surgical biopsy from a metastatic ovarian tumor of high-grade serous histotype, as previously described (3). OVA-BS4 spheroids were isolated from parental OVA-BS4 cells grown under selective culture conditions using the tumor spheroid assay, a well-known method to examine the capacity of tumor cells to grow as multicellular spheroids under non-differentiating and non-adherent conditions, as already published (4).

7. miRNA and gene expression profiles of OVA-BS4 parental and OVA-BS4 spheroid cell lines

Microarray experiments were performed on parental OVA-BS4 and OVA-BS4 spheroids using the commercially available G4851B human whole GE Microarray kit, as previously reported (4) and the G4470B Human miRNA kit (Agilent Technologies), according to manufacturer's instructions.

Table S2. Brescia cohort – microarray data: 73 samples (39 Pt-r vs 34 Pt-s). Equivalence Test. Welch Two samples Test (TOST). equivalence R package. Highlighted: reference miRNA.

miRNA	Mean (sd) log2(exp)		95% CI	p.value[1] log2(exp)
	Pt-r	Pt-s		
**hsa-miR-26a-5p**	**10.29 (0.54)**	10.10 (0.5)	[-0.014, 0.390]	0.080*
hsa-miR-16-5p	10.29 (0.45)	10.91 (0.49)	[-0.260, 0.111]	0.006***

[1] Equivalence range: [-0.36, 0.36]
*<0.10 **<0.05 ***<0.01

Table S3. Brescia cohort - RT-qPCR. Equivalence Test. Welch Two samples Test (TOST). equivalence R package. Highlighted: reference.

miRNA	Mean (sd) Cq		95% CI	p.value[1] Cq
	Pt-r	Pt-s		
**56 samples (29 Pt-r vs 27 Pt-s)**				

**hsa-miR-26a-5p**	24.33 (1.30)	24.60 (1.00)	[-0.788,0.244]	0.194
hsa-miR-16-5p	24.36 (1.19)	24.33 (0.96)	[-0.447,0.520]	0.043**
hsa-miR-191-5p	25.56 (1.06)	25.35 (0.82)	[-0.209,0.634]	0.099*
		**96 samples (48 Pt-r vs 48 Pt-s)**		
hsa-miR-16-5p	24.26 (1.07)	24.35 (0.94)	[-0.433,0.249]	0.016**

[1] Equivalence range: [-0.54, 0.54]
*<0.10 **<0.05 ***<0.01

8. Cell lines and culture conditions

The human ovarian cancer cell line OVCAR3 (RRID: CVCL 0465) was purchased from ATCC. The primary ovarian cancer cell line OSPC2 was established from the ascites of a patient affected by HGSOC progressing after two cycles of a carboplatin-containing regimen (5). Source-patient characteristics are described in Table S4. All cell cultures were confirmed to be free from mycoplasma contamination and OSPC2 was authenticated by short tandem repeat profiling (5). Cells were maintained in RPMI 1640 (Euroclone) supplemented with 10% fetal bovine serum (FBS, Invitrogen), 2 mM glutamine, and penicillin/streptomycin (Euroclone) in a humidified 5% (v/v) CO2 atmosphere at 37°C.

Table S4. Clinical features of the patient and characteristics of the tumor sample used to derive cell line OSPC2

Clinical Parameter	OSPC2
Age at diagnosis (y)	43
Tumor type	adenocarcinoma
Histopathology sub-type	serous
Tumor grade	high
Disease stage	IIIC
Ascites at surgery	yes
CA125 (U/ml) at diagnosis	243
Surgical debulking	sub-optimal
Progression	yes
Death	yes
Cause of death	progression
Overall survival (months)	3
First-line treatment	surgery, carboplatin/paclitaxel
Previous personal history of cancer	no
Year of sampling	2005
Chemotherapy naïve at sample collection	no

9. Cell transfection

Cells were seeded in six-well plates and grown to 30% confluence before transfection. The OSPC2 cell line was transiently transfected with either mirVana™ miR-23-a-3p Inhibitor or mirVana™ miRNA Inhibitor Negative Control (Thermo Fisher Scientific). The OVCAR3 cell line was transiently transfected with either mirVana™ miR-23-a-3p Mimic or mirVana™ miRNA Mimic Negative Control (Thermo Fisher Scientific). The final concentration of miRNA inhibitor, mimic, and negative control was 40 nM. The transfection was performed with Lipofectamine RNAiMAX reagent (Thermo Fisher Scientific). in Opti-MEM medium (Gibco), for 6 hours of incubation, according to the manufacturer's protocol. After transfection, fresh medium with antibiotics was added and cells were grown for 24 hours before drug treatment. Experiments were performed three times.

10. Drug treatment

Carboplatin (Sigma-Aldrich) was dissolved in sterile water at a stock concentration of 10 mg/ml, as reported in the manufacturer's instructions, and stored at 4°C. Different

concentrations of carboplatin were added to cells in each well, 24 hours after transfection. Cells were harvested 48 h post-drug treatment for RNA/protein extraction and cell death assay. Carboplatin IC50 concentrations for OSPC2 and OVCAR3 cell lines were previously tested by our group (5-6).

11. Apoptosis assay

Both floating and adherent cells were collected, washed with PBS and resuspended in a solution of RPMI medium containing Annexin V-Alexa Fluor 647 (Thermo Fisher Scientific) and propidium iodide (PI, Sigma Aldrich). Labelled cells were detected with a flow cytometer (BD FACSCelesta™) and analyzed with BD Diva software (BD Biosciences).

For each cell line and concentration of carboplatin agent, we compared the log2-transformed percentage of total Annexin V+/PI− and Annexin V+/PI+ cells, between scramble- and mimic- or inhibitor- transfected condition, using paired t-test (one-tail).

12. RNA isolation from cell lines and RT-qPCR

Total RNA was extracted from cell lines using TRIzol reagent, according to the manufacturer's protocol. To detect miR-23a-3p expression after transfection, RT-qPCR was performed, as previously described (7).

13. Western blot

Cells were lysed in cold Cell Disruption Buffer (Ambion) containing inhibitors of proteases and phosphatases (Complete and PhosSTOP, Roche). Protein lysates were separated by SDS/PAGE in 4-15% gradient gel (Biorad) and transferred to a nitrocellulose membrane. Blots were saturated in 5% non-fat milk (Euroclone) and incubated with mouse anti-APAF1 antibody (clone 24/Apaf-1, 1:300 dilution, BD Bioscience), followed by incubation with HRP-conjugated anti-mouse (Pierce) at room temperature for 1 h. Immunoreactive bands were detected using LiteAblot Turbo (Euroclone) chemiluminescence reagent and a digital imager (Cambridge UVITEC). The blots were then incubated with rabbit anti-GAPDH antibody (clone GT239, 1:10,000 dilution, Genetex) followed by HRP-anti-rabbit antibody (Pierce) and developed as described above.

References

1. Calura E, Paracchini L, Fruscio R, DiFeo A, Ravaggi A, Perrone J, et al. A prognostic regulatory pathway in stage I epithelial ovarian cancer: new hints for the poor prognosis assessment. Ann Oncol. 2016; 27(8): 1511-19.
2. Bignotti E, Calza S, Tassi RA, Zanotti L, Bandiera E, Sartori E, et al. Identification of stably expressed reference small non-coding RNAs for microRNA quantification in high-grade serous ovarian carcinoma tissues. J Cell Mol Med. 2016; 20(12): 2341-48.
3. Santin AD, Zhan F, Bellone S, Palmieri M, Cane S, Bignotti E, et al. Gene expression profiles in primary ovarian serous papillary tumors and normal ovarian epithelium: identification of candidate molecular markers for ovarian cancer diagnosis and therapy. Int J Cancer. 2004;112:14–25.
4. Zanotti L, Romani C, Laura Tassone L, Todeschini P, Tassi RA, Bandiera E, et al. MAL gene overexpression as a marker of high-grade serous ovarian carcinoma stem-like cells that predicts chemoresistance and poor prognosis. BMC Cancer 2017 May 25;17(1):366.
5. Tassi RA, Todeschini P, Siegel ER, Calza S, Cappella P, Ardighieri L, et al. FOXM1 expression is significantly associated with chemotherapy resistance and adverse prognosis in non-serous epithelial ovarian cancer patients. J Exp Clin Cancer Res. 2017; 36(1):63.

6. Romani C, Capoferri D, Grillo E, Silvestri M, Corsini M, Zanotti L, et al. The Claudin-Low Subtype of High-Grade Serous Ovarian Carcinoma Exhibits Stem Cell Features. Cancers (Basel). 2021; 13(4):906.
7. Todeschini P, Salviato E, Paracchini L, Ferracin M, Petrillo M, Zanotti L, et al. Circulating miRNA landscape identifies miR-1246 as promising diagnostic biomarker in high-grade serous ovarian carcinoma: A validation across two independent cohorts. Cancer Lett. 2017; 388: 320-7.
