# Supplementary material for: Comprehensive Profiling of Hypoxia-Related miRNAs Identifies miR-23a-3p Overexpression as a Marker of Platinum Resistance and Poor Prognosis in High-Grade Serous Ovarian Cancer
Source: Cancers (Basel). 2021 Jul 4;13(13):3358. doi: 10.3390/cancers13133358 (PMC8268862; doi:10.3390/cancers13133358)
Supplement: Supplementary file 1 [file cancers-13-03358-s001.zip › Todeschini_etal_SupplResults_Cancers.pdf]

Supplementary Results

Table S1. Hypoxia-related miRNAs selected for expression analysis.

Candidate miRNAs	Reference
miR-21-3p, miR-23a-3p, miR-23b-3p, miR-24-3p, miR-26a-5p, miR-26b-5p, miR-27a-3p, miR-30b-5p, miR-93-5p, miR-103a-3p, miR-106a-3p, miR-107, miR-181a-5p, mir-181b-5p, miR-181c-5p, miR-192-5p, miR-195-5p	Kulshreshtha R. et al. (9)
miR-210	Kulshreshtha R. et al. (9) and Giannakakis A. et al. (11)
miR-21 (previous ID miR-21-5p)	Kulshreshtha R. et al. (9) and Polytarchou C. et al. (12)
miR-145-5p (previous ID miR-145)	Qing X. et al. (13)
miR-199a-5p (previous ID miR-199a)	Jun H. et al. (14) and Hemant P.J. et al. (16)
miR-125b-5p (previous ID miR-125b)	Kulshreshtha R. et al. (9) and Jun H. et al. (14)
miR-138	Yeh Y.M. et al. (15)
miR-199a-3p	Kinose Y. et al. (17)
miR-630	Rupaimoole R. et al. (18)

Table S2. Overall survival analysis.Univariate and multivariate Overall Survival (OS) analysis (Coxph) for miR-23a-3p and miR-181c-5p expression in the Brescia and TCGA cohorts.

miRNA-name	UNIVARIATE			MULTIVARIATE[1]		
	Hazard	SE	p-value	Hazard	SE	p-value
**Brescia Cohort – microarray data (95 samples)**						
hsa-miR-23a-3p	1.503	0.236	0.085*	1.355	0.232	0.190
hsa-miR-181c-5p	1.208	0.117	0.107	1.130	0.119	0.306
**TCGA Cohort – RNA-seq data (178 samples)**						
hsa-miR-23a-3p	1.155	0.143	0.314	1.159	0.144	0.306
hsa-miR-181c-5p	0.877	0.090	0.145	0.964	0.094	0.693

[1] Multivariate model accounted for age and residual tumor (2 classes: RT=0 and RT>0).

*<0.10 **<0.05 ***<0.01

Table S3. Complete results of Multivariate Survival Analysis (Coxph) of the Brescia cohort, reported in Table 5 of the main text. miRNA expression in 145 samples was evaluated by RT-qPCR.

miRNA-name	Residual tumor[2]			Age			Bevacizumab[3]			miRNA -ΔCq[1]		
	Hazard	SE	p-value	Hazard	SE	p-value	Hazard	SE	p-value	Hazard	SE	p-value
**Overall Survival**												
miR-23a-3p	2.271	0.266	0.002***	1.036	0.009	<0.001***	0.863	0.349	0.672	1.195	0.077	0.021**
miR-181c-5p	2.322	0.265	0.001***	1.031	0.009	0.001***	0.944	0.350	0.870	1.025	0.045	0.587
**Progression-Free Survival**												
miR-23a-3p	2.554	0.229	<0.001***	1.021	0.008	0.011**	0.550	0.267	0.025**	1.244	0.077	0.004***
miR-181c-5p	2.568	0.229	<0.001***	1.014	0.008	0.072*	0.611	0.267	0.065*	1.040	0.041	0.346

[1] reference miR-16-5p

[2] 2 classes: RT>0 versus RT=0

[3] 2 classes: yes versus no

*<0.10 **<0.05 ***<0.01. Highlighted: significant.

Table S4. Multivariate Progression-Free Survival Analysis (Coxph) of the Brescia cohort. miRNA expression, stratified into three groups based on quartiles of the -ΔCq distribution (Q_1: low, n=37; Q_2-Q_3: moderate, n=72; Q_4: high, n=36), evaluated in 145 samples by RT-qPCR.

Variable	HR	SE	p-value
Reference group: low (miR-23a-3p[1] ≤ -1.46)			
Residual Tumor (RT=1)	2.647	0.234	**<0.001** ***
Bevacizumab (yes)	0.540	0.271	**0.023** **
Age	1.018	0.008	**0.023** **
Moderate (-1.46 < miR-23a-3p[1] ≤ 0.07)	1.868	0.235	**0.007** ***
High (miR-23a-3p[1] > 0.07)	1.918	0.273	**0.017** **

[1] reference miR-16-5p

*<0.10 **<0.05 ***<0.01.

Table S5. Multivariate Survival Analysis (Coxph) of the subgroup of 22 patients treated with bevacizumab in the Brescia cohort. miRNA expression was evaluated by RT-qPCR.

miRNA-name	Residual tumor			Age			miRNA ΔCq[1]		
	Hazard	SE	p-value	Hazard	SE	p-value	Hazard	SE	p-value
**Overall Survival**									
miR-23a-3p	3.099	0.710	0.111	0.996	0.036	0.911	1.145	0.372	0.716
miR-181c-5p	2.810	0.752	0.170	0.998	0.036	0.950	0.900	0.266	0.693
**Progression Free Survival**									
miR-23a-3p	8.048	0.695	**0.003*****	0.933	0.029	0.017**	1.836	0.311	0.051*
miR-181c-5p	7.400	0.715	**0.005*****	0.939	0.029	0.030**	1.293	0.200	0.199

[1] reference miR-16-5p

*<0.10 **<0.05 ***<0.01. Highlighted: significant.

miR-23a-3p/APAF1 axis and carboplatin sensitivity

To functionally assess the miR-23a-3p-APAF1 interaction, we performed some pilot functional studies in two HGSOC cells lines, which expressed high (OSPC2) or low (OVCAR3) miR-23a-3p levels, as detected by RT-qPCR and reported in Figure S1. Transient inhibition of miR-23a-3p in OSPC2 cells (Figure S2A) resulted in an increase in APAF1 protein expression levels (Figure S3A). Conversely, the transient induction of miR-23a-3p in OVCAR3 cells (Figure S2B) caused a reduction in APAF1 protein levels (Figure S3B).

We next tested the sensitivity of the transfected cells to treatment for 48 hours with different concentrations of carboplatin.

As reported in Figure S3C and Table S6, inhibition of miR-23a-3p expression in OSPC2 cells led to a significant increase in apoptosis induced by 300 μM carboplatin, compared cells transfected with the scramble control (p-value = 0.021). Moreover, forced expression of miR-23a-3p in OVCAR3 cells (Figure S3B) significantly reduced the percentage of apoptotic cells, compared with the control-transfected cells (Figure S3D and Table S6), increasing cell resistance to carboplatin treatment at 30 and 35 μM (p-value = 0.013 and 0.016, respectively). Overall, these observations support the hypothesis that APAF1 expression regulation, mediated by miR-23a-3p, affects HGSOC cell sensitivity to carboplatin.

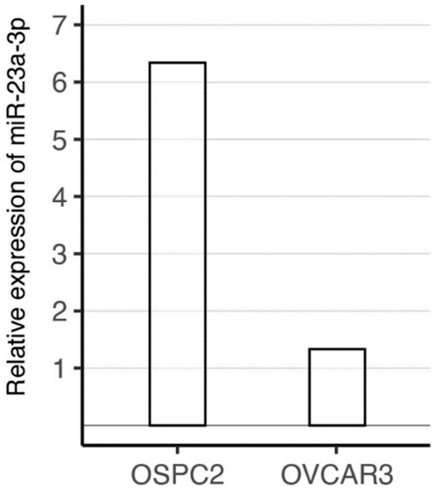

Figure 1. miR-23a-3p expression in HGSOC cell lines by RT-qPCR. OSPC2 cells showed higher levels of miR-23a-3p expression compared to OVCAR3 cells. miR-23a-3p levels were normalized to the geometric average expression of U6 and miR-16-2-3p as references.

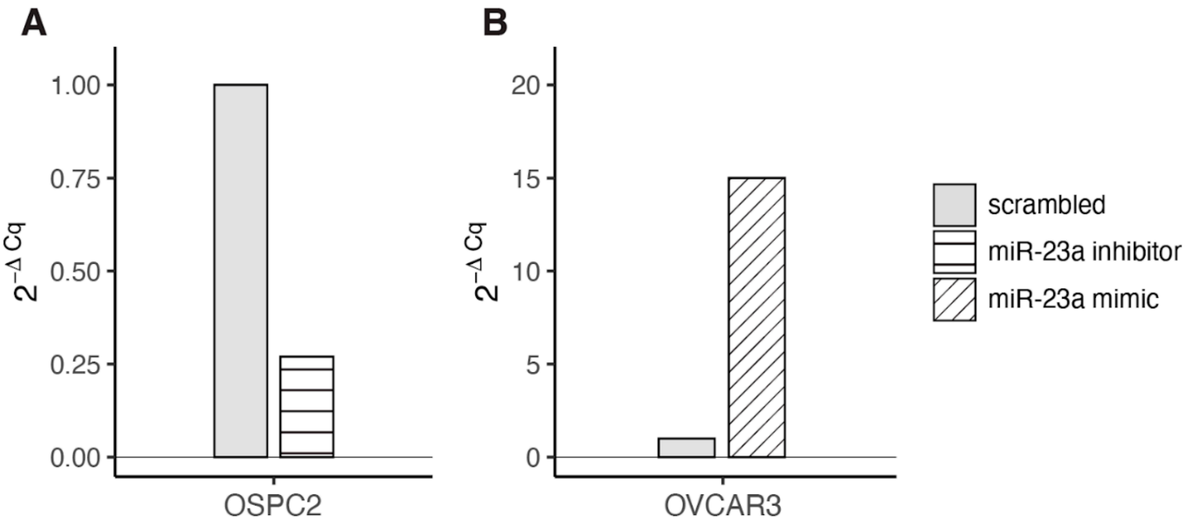

Figure S2. Boxplots of a representative experiment showing miR-23a-3p expression modulation, after miR-23a-3p (**A**) silencing with inhibitor (horizontal lines) or (**B**) mimic transfection (diagonal lines), compared to scramble-miR control (gray bar). Levels were measured by RT-qPCR and normalized to the geometric average expression of U6 and miR-16-2-3p as references.

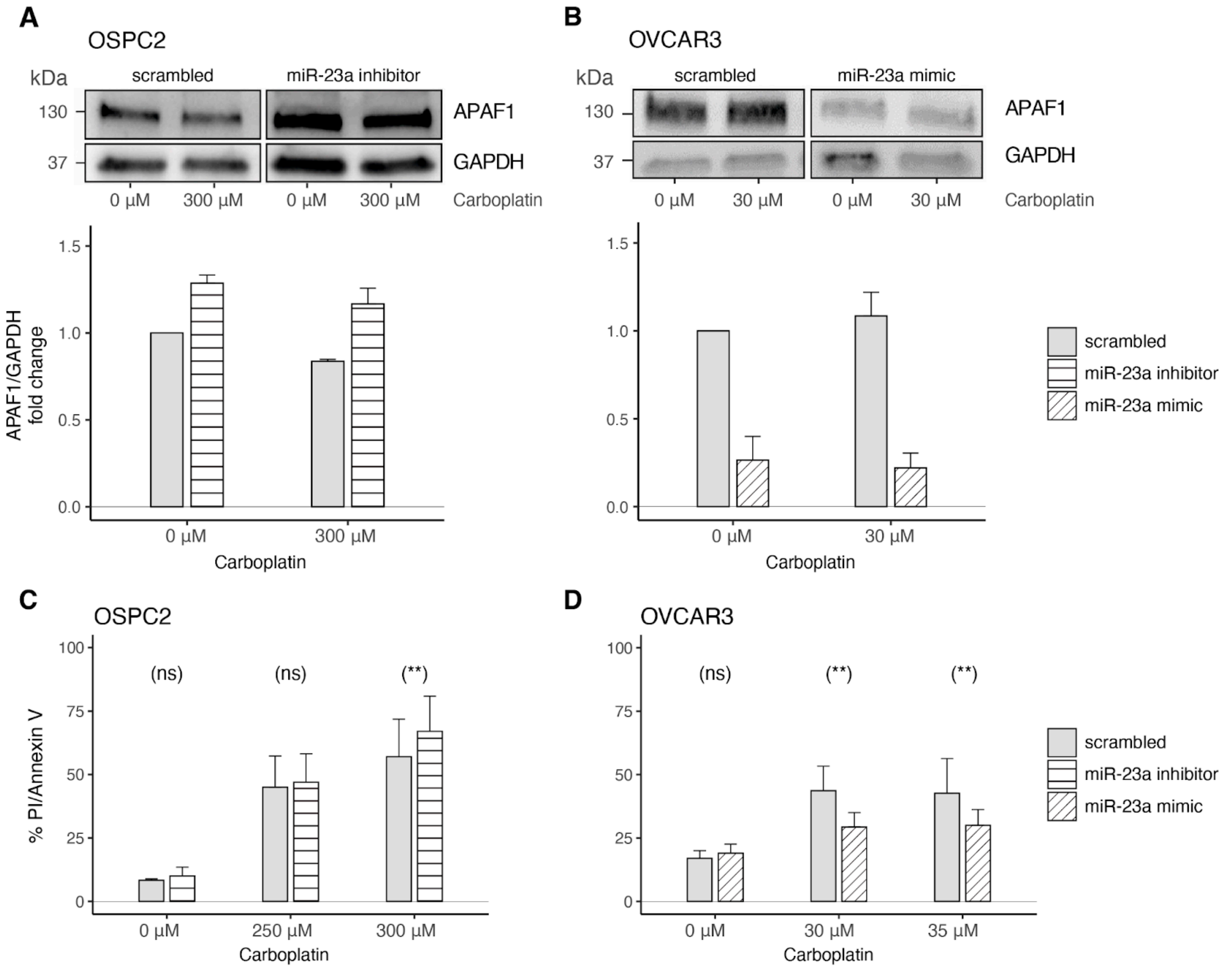

Figure S3. Effects of miR-23a-3p on APAF1 expression and response to carboplatin in OSPC2 and OVCAR3 cells. Panel A and B show images from representative immunoblots and changes in APAF1 protein levels normalised against GAPDH. Panels C and D show percentages of apoptotic cells [Anneexin V and PI] in cells subjected to the indicated treatments. Plotted are means (± standard deviation) from 3 (OSPC2) or 4 (OVCAR3) experiments independent. One-tailed t-test: (ns) not significant, (*) p≤0.05, (**) p≤0.025, (***) p≤0.01.

Table S6. Apoptotic cell death after carboplatin treatment in OSPC2 and OVCAR3 cells transfected with miR23a-3p inhibitor or miR-23a-3p mimic vs scramble-miRNA control.

Carboplatin concentration µM	% Apoptotic Cell Death		T test[1]	
	Inhibitor/Mimic-transfected Mean (sd)	Scramble-miRNA control Mean (sd)	Statistic	p-value log2(diff)
	OSPC2	miR-23a-3p inhibitor (n=3)		
No treatment	10.0 (3.5)	8.3 (0.6)	-0.50	0.333
250	47.0 (11.1)	44.0 (12.3)	-1.27	0.167
300	67.0 (12.9)	57.0 (14.8)	-4.73	0.021**
	OVCAR3	miR-23a-3p mimic (n=4)		
No treatment	19.0 (3.6)	17.0 (3.0)	-1.21	0.825
30	29.3 (5.7)	43.7 (9.7)	6.13	0.013**
35	30.0 (6.2)	42.7 (13.6)	5.55	0.016**

[1] paired, one-tail. *<0.05, **<0.025, ***<0.01.
